# Hepatocellular carcinoma: signaling pathways, targeted therapy, and immunotherapy

**DOI:** 10.1002/mco2.474

**Published:** 2024-02-04

**Authors:** Xiaoting Luo, Xin He, Xingmei Zhang, Xiaohui Zhao, Yuzhe Zhang, Yusheng Shi, Shengni Hua

**Affiliations:** ^1^ Department of Radiation Oncology Zhuhai People's Hospital Zhuhai Hospital Affiliated with Jinan University Zhuhai China; ^2^ Guangdong Provincial Key Laboratory of Tumor Interventional Diagnosis and Treatment Zhuhai People's Hospital Zhuhai Hospital Affiliated with Jinan University Zhuhai China; ^3^ Department of Neurobiology School of Basic Medical Sciences Southern Medical University Guangzhou China

**Keywords:** hepatocellular carcinoma, immunotherapy, signaling pathways, targeted therapy

## Abstract

Hepatocellular carcinoma (HCC) is the most common primary liver cancer with a high mortality rate. It is regarded as a significant public health issue because of its complicated pathophysiology, high metastasis, and recurrence rates. There are no obvious symptoms in the early stage of HCC, which often leads to delays in diagnosis. Traditional treatment methods such as surgical resection, radiotherapy, chemotherapy, and interventional therapies have limited therapeutic effects for HCC patients with recurrence or metastasis. With the development of molecular biology and immunology, molecular signaling pathways and immune checkpoint were identified as the main mechanism of HCC progression. Targeting these molecules has become a new direction for the treatment of HCC. At present, the combination of targeted drugs and immune checkpoint inhibitors is the first choice for advanced HCC patients. In this review, we mainly focus on the cutting‐edge research of signaling pathways and corresponding targeted therapy and immunotherapy in HCC. It is of great significance to comprehensively understand the pathogenesis of HCC, search for potential therapeutic targets, and optimize the treatment strategies of HCC.

## Graphical abstract

This review describes the signaling pathways and the clinical targeted therapies and immunotherapy in hepatocellular carcinoma (HCC). Abnormal activation of signaling pathways in HCC can lead to a series of physiological activities such as cell proliferation, metabolism, metastasis, angiogenesis, activated stemness and immune evasion. Therefore, corresponding targeted drugs and immune checkpoint inhibitors in HCC have been developed and put into clinical use.

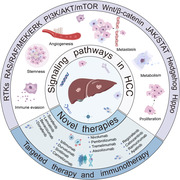

## 1 INTRODUCTION

According to the 2020 global cancer statistics data from the World Health Organization (WHO), liver cancer ranks sixth in incidence and third in mortality among all cancers.^1^ Around 90% of all primary liver cancers are hepatocellular carcinoma (HCC).^2^, ^3^ The main risk factors are chronic viral hepatitis due to hepatitis B virus (HBV) and hepatitis C virus (HCV) infections, aflatoxin poisoning, chronic alcohol intake, diabetes mellitus, and nonalcoholic fatty liver disease.^4^, ^5^ Due to the high degree of malignancy and insidious onset, most patients are diagnosed in the middle or late stages, thereby losing the optimal opportunity for treatment. Traditional therapies, including hepatectomy, liver transplantation, transcatheter arterial chemoembolization (TACE), and chemotherapy, have poor outcomes for advanced HCC patients.^6^, ^7^

The mechanism underlies both the development of HCC as well as its progression is still not fully understood.^8^ HCC is the result of accumulation of genetic mutations and epigenetic modifications of proto‐oncogenes and associated driver genes, and is characterized by molecular heterogeneity.^9^, ^10^ Researchers have long been committed to the study of molecular mechanism involved in hepatocarcinogenesis. On the basis of this research results, targeted therapy and immunotherapy has provided new methods for the clinical treatment of HCC especially advanced HCC patients.^11^, ^12^, ^13^ In 2007, the tyrosine kinase inhibitor (TKI) sorafenib was approved,^14^ and more targeted drugs were applied to the clinical treatment of HCC.^15^, ^16^ In 2013, the first clinical trial of immunotherapy showed that tremelimumab, a cytotoxic T‐lymphocyte protein 4 (CTLA‐4) inhibitor, improved objective response rates (ORRs) in HCC patients with HCV infection.^17^ Subsequently, the immune checkpoint inhibitors (ICIs) that antiprogrammed cell death protein 1 (PD‐1), CTLA‐4 have increasingly been used for the treatment of HCC.^18^, ^19^ At present, the combination of targeted drugs and ICIs has shown good efficacy and safety. However, as targeted therapy progresses, so does drug tolerance.

In order to overcome drug resistance and develop novel and effective targeted therapy drugs, to explore the optimal combination approach for systemic therapy, a comprehensive understanding of HCC‐related molecular signaling pathways and corresponding targeted therapy is critical. This review provides a detail overview of the major oncogenic molecular signaling pathways in HCC, including receptor tyrosine kinases (RTKs), RAS/RAF/MEK/extracellular‐signal‐regulated kinase(ERK), phosphoinositide 3‐kinase (PI3K)/AKT/mammalian target of rapamycin (mTOR), Wnt/β‐catenin, janus‐kinase (JAK)/signal transducer activator of transcription (STAT), Hedgehog (Hh), Hippo pathway, and related targeted therapy drugs. Furthermore, we also summarize the main immune‐related molecular mechanisms and corresponding immunotherapy in HCC. This paper still aims to provide new directions for HCC targeting and immunotherapy and explore optimal combination treatment strategies.

## 2 RELATED SIGNALING PATHWAYS IN HCC

The dysregulation of cellular signals transduction leads to the occurrence and progression of HCC. In‐depth understanding of HCC‐related signaling pathways is useful for cancer diagnosis and the discovery of potential therapeutic targets. The common signaling pathways associated with HCC include RTKs, RAS/RAF/MEK/ERK, PI3K/AKT/mTOR, Wnt/β‐catenin, JAK/STAT, Hh, and Hippo pathways. RTKs are a group of cell membrane receptors that play a critical role in HCC progression. RTKs inhibitors are the earliest HCC‐targeted drugs used clinically. The RAS/RAF/MEK/ERK pathway is regulated by upstream RTKs, and it is related to the poor prognosis of HCC patients. The PI3K/AKT/mTOR pathway can promote cell proliferation and tumor metastasis. It is also one of the most frequently activated signaling pathways in HCC and is the main mechanism of HCC therapy resistance. Abnormal activation of Wnt/β‐catenin pathway molecules is usually found in advanced HCC. It involves in the regulation of tumor migration, invasion, stem cells maintenance, and so on. The JAK/STAT pathway is also regulated by RTKs, it drives the occurrence and development of HCC by modulating cell proliferation, angiogenesis, and cellular metabolism. At the same time, this pathway involves in the regulation of immune microenvironment, which is expected to provide a new strategy for targeted and immunotherapy combination in HCC. The signaling molecules related to Hh pathway are usually overexpressed in HCC, and this pathway is associated with tumor metastasis and chemotherapy resistance. The Hippo pathway participates in HCC progression by regulating cell proliferation, apoptosis and stem cell self‐renewal. It provides new targets for current HCC research.

### 2.1 RTKs pathway

RTKs are the largest class of enzyme linked receptors, they acting as receptors can be activated by binding to corresponding ligands, and can also act as kinase to phosphorylate the tyrosine residues of the corresponding target proteins to activate downstream signaling pathway.^20^ The RTKs signaling pathway related to the development of HCC mainly include vascular endothelial growth factor receptor (VEGFR), fibroblast growth factor receptor (FGFR), epidermal growth factor receptor (EGFR), hepatocyte growth factor receptor (HGFR), platelet‐derived growth factor receptor (PDGFR), and insulin‐like growth factor receptor (IGFR). Through multilevel regulation and cascaded amplification, they can affect downstream signaling pathway, such as mitogen‐activated protein kinases (MAPK) and PI3K/AKT.^21^ Almost all types of RTKs are targeted by antitumor drugs currently developed, especially drugs targeting VEGFR and EGFR, which have been put into first line clinical use.^22^, ^23^ What follows is a detailed introduction of different kinds of RTKs, as well as their signaling pathway activation mechanisms, biological functions in HCC, and the related inhibitors.

#### 2.1.1 VEGFR

Primary liver cancer is rich in blood, and blood vessels play a significant part in the development and metastasis of malignant tumors. Solid tumors cannot grow to > 2−3 mm^3^ without additional blood vessel support. Vascular endothelial growth factor (VEGF) with high specificity can increase vascular permeability and promote angiogenesis, thereby promoting the growth, invasion, and metastasis of HCC.^24^

**FIGURE 1:**
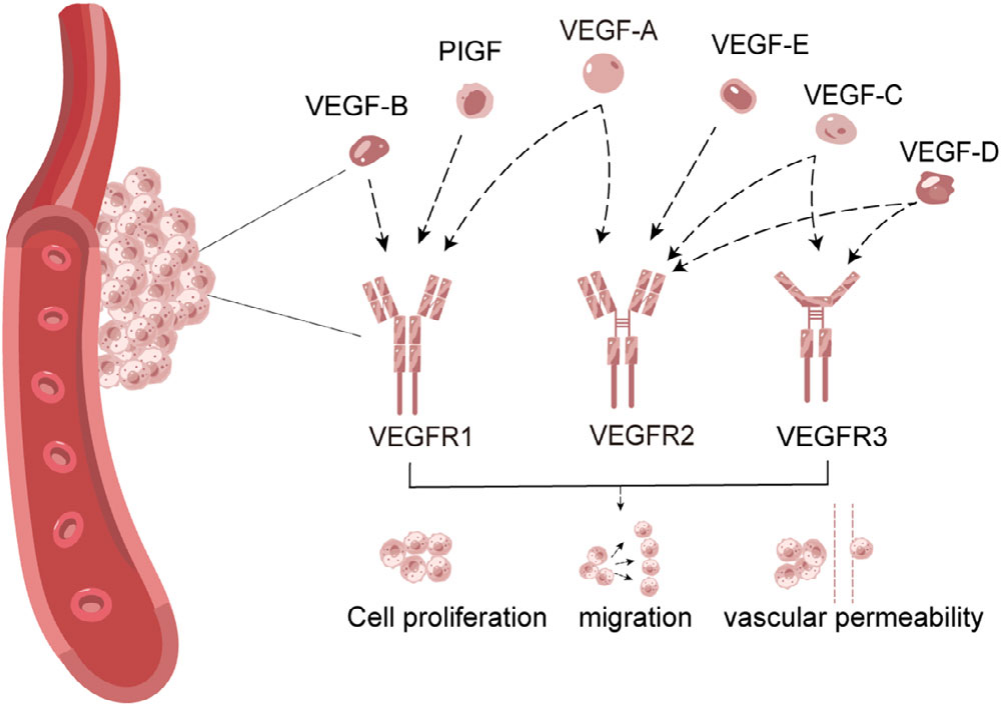
VEGF protein family and receptor signaling pathway. A family of VEGF includes VEGF‐A, VEGF‐B, VEGF‐C, VEGF‐D, VEGF‐E, and PIGF. The high‐affinity receptors that specifically bind to VEGF are called VEGFR, which are mainly divided into VEGFR‐1, VEGFR‐2, and VEGFR‐3. VEGF signaling pathway can induce endothelial cell proliferation, migration, and increase vascular permeability, and then participate in the occurrence and progression of angiogenesis‐dependent diseases. PIGF, placental growth factor; VEGF, vascular endothelial growth factors; VEGFR, vascular endothelial growth factor receptors;.

The VEGF family mainly includes VEGF‐A, B, C, D, and E. VEGF‐A is directly referred to VEGF, which plays a leading role in regulating angiogenesis and promoting tumor growth. VEGF‐B works in non‐neovascularized tumors, while VEGF‐C and VEGF‐D are mainly involved in tumor lymphangiogenesis. VEGF‐E also has a proangiogenic function. The corresponding VEGF receptors are divided into three main subtypes: VEGFR1, VEGFR2, and VEGFR3.^25^ VEGFR1 has the ability to bind with VEGF‐A, VEGF‐B, and placental growth factor (PIGF). VEGFR2 can bind with VEGF‐A, VEGF‐C, VEGF‐D, and VEGF‐E. VEGFR3 is the receptor for VEGF‐C and VEGF‐D (Figure 1).^26^, ^27^

VEGFR1 has a role in the control of hematopoiesis, the expression of VEGFR1 is influenced by the microenvironment, and activation of VEGFR1 can have an effect on vascular permeability as well as proangiogenic mediators.^28^ VEGFR1 is crucial for the tissue‐specific release of growth factors from hepatic sinusoidal endothelial cells and works to prevent hepatocellular injury caused by hepatotoxic substances.^29^ VEGFR2 is expressed in almost all endothelial cells and is the most critical factor for promoting angiogenesis. VEGFR2 mediates most of the major downstream effects of VEGF and is involved in enhancing vascular permeability and promoting endothelial cell proliferation, invasion, migration, as well as angiogenesis. VEGFR3 activation induces lymphangiogenesis and modulates lymphatic vessel formation.^30^, ^31^

The hypoxic microenvironment of HCC can promote the generation of growth factors including insulin‐like growth factor 2 (IGF2), hypoxia‐inducible factors (HIF), and so on. This can further stimulate the expression of VEGF and other growth factors, enhance tumor angiogenesis, and cause the growth and metastasis of tumor.^32^ Research has shown that VEGF is highly expressed in patients with chronic liver disease who are infected with HBV. VEGF mRNA is overexpressed in most HCC tissues, and its expression is associated with the invasiveness, vessel density, metastasis, recurrence, and prognosis of HCC patients. In addition, increased expression of VEGF and VEGFR has been detected in HCC cell lines and tissues, as well as in the serum of HCC patients. Simultaneously, inhibition of the VEGF–VEGFR‐related signaling pathway has been shown to significantly hamper the proliferation, invasion, and migration of HCC.^33^, ^34^, ^35^ The majority of medications used in the systemic treatment of HCC patients target VEGF and VEGFR.

#### 2.1.2 EGFR

EGFR belongs to the RTKs Erb family. EGFR is a transmembrane glycoprotein composed of extracellular ligand‐binding and cytoplasmic tyrosine kinase domains. Its physiological function is the regulation of epithelial tissue development and homeostasis. The combination of extracellular signals and EGFR regulates cell survival, proliferation, differentiation, and motility.^36^, ^37^, ^38^ EGFR signaling is involved in fatty acid biosynthesis,^39^ glucose catabolism,^40^ and other metabolic processes, thereby regulating cancer cell proliferation.

EGFR has six ligands, including epidermal growth factor (EGF), epiregulin (EREG), transforming growth factor‐alpha (TGF‐α), amphiregulin (AREG), beta‐cellulin, and heparin‐binding EGF (HB‐EGF). EREG is poorly expressed in most normal tissues, but overexpressed EREG triggers its downstream signaling pathway and promotes tumor progression while binding to EGFR.^41^ HB‐EGF promotes HCC proliferation, invasion, and angiogenesis.

HB‐EGF inhibitors can be used in combination with sorafenib for individualized HCC patients.^42^ Most HCC cell lines exhibit high EGFR expression levels, and a clinical study conducted at Shanghai Renji Hospital (ClinicalTrials.gov, NCT04642547) suggested that lenvatinib in combination with gefitinib may be particularly beneficial for HCC patients with high EGFR expression level.^43^ The first‐ and second‐ generation of EGFR‐TKI such as gefitinib, afatinib, and osimertinib significantly prolong the survival time of patients with EGFR‐activated tumors.^44^, ^45^, ^46^

#### 2.1.3 FGFR

Fibroblast growth factors (FGFs), named for their mitogenic ability of fibroblasts, often mediate metabolic function, tissue repair, and mature tissues regeneration by reactivating signaling pathways.^47^ The FGF family consists of almost 20 ligands, which mainly exert their effects by binding to high‐affinity tyrosine kinase receptors encoded by four genes (FGFR1, FGFR2, FGFR3, and FGFR4) and activating downstream signaling pathways.^48^

The FGF/FGFR signaling pathway is involved in regulating tumor cellular function, such as cancer cell proliferation, angiogenesis, therapeutic response, as well as tumor metastasis.^49^ As an effective mitogen, FGF2 plays an important role in the development and progression of HCC. This molecule stimulates DNA synthesis in HCC cells, promotes tumor growth and invasion, and participates in angiogenesis. In addition, FGF2 was found to upregulate the expression of VEGFA in majority of cell types, including HCC cells.^50^, ^51^, ^52^, ^53^ FGF19 is also overexpressed in HCC cells,^54^, ^55^, ^56^ and its expression can serve as a biomarker for the detection of HCC and the prognostication of therapeutic response.^55^ FGF19 and its receptor FGFR4, and the activated signaling pathways, including EGFR, Wnt/β‐catenin, ERK, and STAT3/IL‐6, may also be new targets for HCC treatment.^56^

It has been reported that FGFR3 and FGFR4 levels are elevated in HCC cells. Highly selective FGFR4 inhibitors like H3B‐6527 can greatly slow down the growth of HCC cell and can be used in patients with mutations in FGF19‐related signaling pathways.^57^ In addition, the FGFR1‐3 inhibitor infigratinib and the FGFR4 inhibitor FGF401 were able to effectively disrupt signaling pathways that play a key role in cell proliferation, survival, metastasis, and drug resistance, thus exhibiting potent antitumor activity in tumor models of HCC with FGFR2/3 or FGF19/FGFR4 high‐expression.^58^

#### 2.1.4 HGFR

Hepatocyte growth factor (HGF) was originally identified as a potent mitogen for hepatocyte. It is a natural endogenous ligand for the MET receptor to stimulate hepatocyte proliferation.^59^, ^60^ The combination of HGF with its receptor c‐Met would activate downstream signaling pathways including JAK/STAT3, PI3K/AKT/NF‐κB, and RAS/RAF. HGF/c‐Met has been reported to exert several functions such as inflammatory response, liver fibrosis, and accelerated development of HCC.^60^ HGF stimulation promotes the Warburg effect and glutamine breakdown, thereby promoting the biogenesis of multiple cancer cells. c‐Met is a type of RTK consisting of disulfide‐bonded heterodimer complexes. Increased c‐Met activity initiates, drives, and facilitates the progression of HCC.^61^ c‐Met may also stimulate the expression of VEGF‐A, which boosts the angiogenesis of tumor.^62^ Research shows that enhanced c‐Met transcription and c‐Met expression in HCC promote cell proliferation, regeneration, and survival.^63^ The HGF/c‐Met is considered as biomarker of poor prognosis and tumor aggressiveness in HCC patients.^64^

MET‐targeted inhibitor and autophagy inhibitor had a good effect on inhibiting the development of HCC.^65^ It has been reported that HGF overexpression and MET activation can be detected in sorafenib‐resistant HCC cells.^66^ Further studies demonstrated that HCC cells with HGF stimulation are resistant to sorafenib, while inhibiting ERK and STAT3 expression can eliminate the effect of HGF by decrease the levels of Snail. Regorafenib reversed HGF‐induced sorafenib resistance through inhibiting ERK and STAT3.^67^ Zhang et al.^68^ found that E‐twenty‐six transformation‐specific variant 1 (ETV1) expression was elevated in HCC specimens, and HGF upregulated ETV1 expression by activating the c‐Met‐ERK1/2‐ELK1 pathway, thereby promoting HCC metastasis. The combination of the protein tyrosine kinase 2 (PTK2) inhibitor defactinib and the c‐Met inhibitor capmatinib significantly inhibits ETV1‐induced HCC metastasis.^68^ It has been reported that the down‐regulated Cytochrome P450 (CYP1A2) in HCC can induce upregulation of HIF‐1α and synthesis of HGF, thus activate signaling of MET/PI3K/AKT/NF‐κB, thereby promoting tumor growth, migration, and invasion, suggesting that CYP1A2 may be a tumor inhibitor and a new independent prognostic marker for HCC patients.^69^ Although HGF/c‐Met‐targeted therapies have made breakthroughs in the treatment of some cancers, HGF/c‐Met‐targeted monotherapy has failed to show significant clinical efficacy in most cancers.^70^

#### 2.1.5 IGFR

The complex interplay between cancer cells and their microenvironments plays an important role in cancer progression. The release of insulin‐like growth factors (IGFs) by the tumor microenvironment (TME) and cancer cells supports neovascularization, proliferation, maintenance, and migration of cancer cells.^71^ IGFs specifically bind to three surface receptors, IGF‐1R, IGF‐2R, and insulin receptor (IR). It has been found that IGF‐1R synthesis is increased and IGF‐2 expression is abnormal during the development of human HCC.^72^ IGF‐1 participates in the incidence and progression of cancer by inducing autophagy. The increasing expression of IGF‐1 is closely related to poor prognosis of HCC patients.

The biological activities of IGF‐1 and IGF‐2 can be regulated by IGF‐binding proteins (IGFBPs). IGFBP‐3 inhibits the transcription of EGR1 and its target genes bFGF and PDGF by inhibiting IGF‐1‐dependent activation of ERK and AKT. This suggests IGFBP‐3 plays an important role in regulating the proliferation of HCC cell and that IGFBP‐3 may be a target for the treatment of HCC.^73^ BTB and CNC homolog 1 (BACH1) is a fundamental leucine zipper transcription factor that regulate oxidative stress, immunity, hematopoiesis, cell cycle, and heme homeostasis. Recent studies have shown that BACH1 facilitates the growth and metastasis of HCC by upregulating genes associated with cancer progression, including IGF‐1R and PTK2. In addition, linsitinib, an IGF‐1R inhibitor, has been found to significantly inhibit the growth and metastasis of HCC mediated by BACH1 when used in combination with defactinib, a PTK2 inhibitor.^74^, ^75^ The antioxidant molecule vitamin D suppresses the growth of HCC cells by silencing IGF‐1 receptor signaling.^76^ Furthermore, experimental studies have shown that IGF and FGF signaling are partly responsible for acquired resistance to sorafenib treatment in patients with HCC, and restraining the activation of these pathways will benefit some patients who progress after treatment with sorafenib.^77^

#### 2.1.6 PDGFR

**FIGURE 2:**
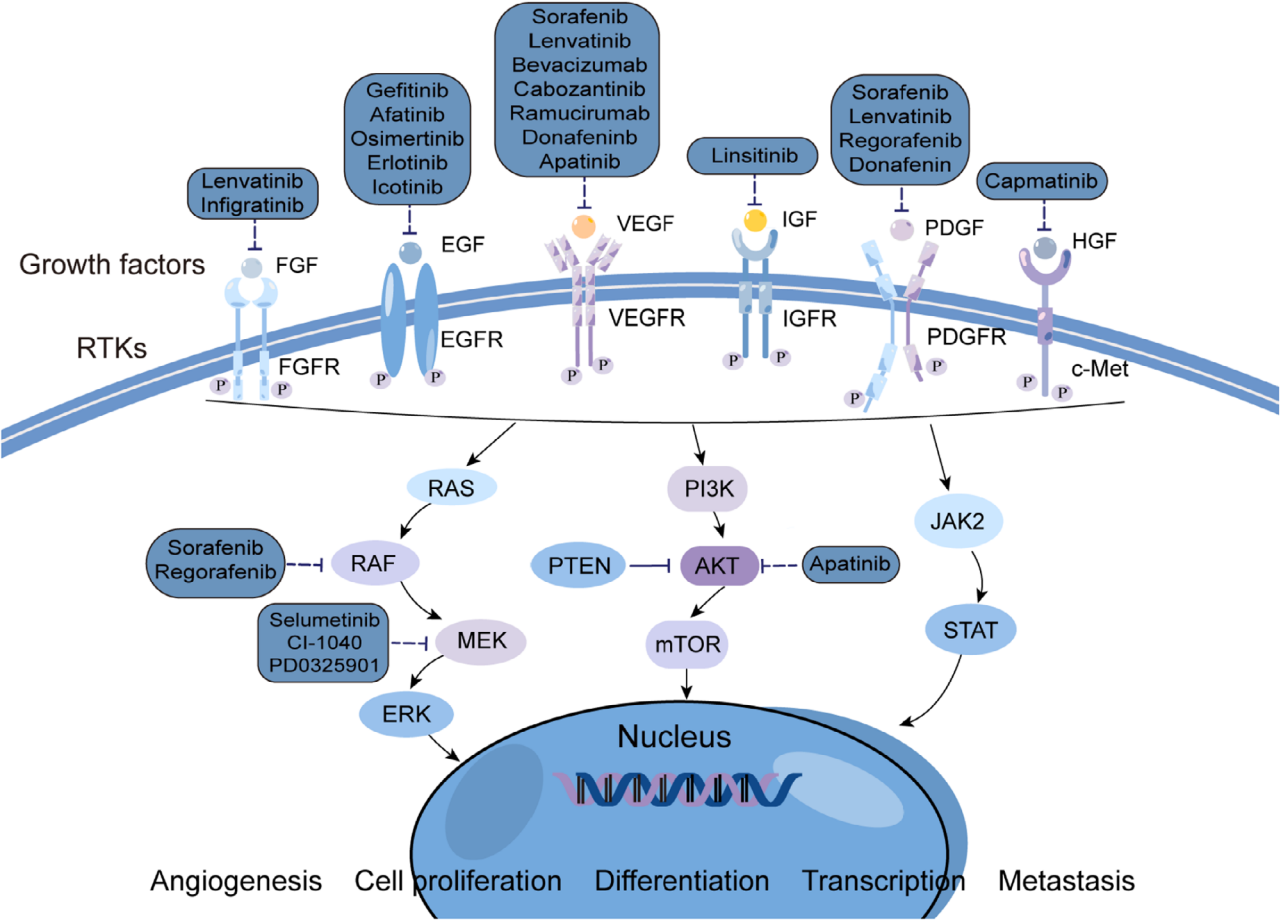
RTKs and its related downstream signaling pathways and their targeted therapies for hepatocellular carcinoma. The binding of RTKs to their corresponding growth factors can activate their protein kinase function, thereby regulating the downstream signal transduction pathways, expanding signal transduction and activating a series of biochemical reactions in cells. RTKIs are a hot spot in the research and development of antitumor drugs. At present, a variety of RTKIs related to HCC have entered phases I–III clinical trials and clinical use. EGFR, epidermal growth factor receptor; FGFR, fibroblast growth factor receptor; HGF, hepatocyte growth factor;c‐Met, cellular mesenchymal epithelial transition factor; IGFR, insulin‐like growth factor receptor; PDGFR, platelet‐derived growth factor receptor; RTKs, receptor tyrosine kinases; RTKIs, receptor tyrosine kinase inhibitors; PTEN, phosphatase and tensin homolog; PI3K, phosphoinositide 3 kinase

There are two subtypes of the PDGFR, PDGFRα and PDGFRβ, which are encoded by two distinct genes, PDGFRA and PDGFRB.^78^ Platelet‐derived growth factor (PDGF) is involved in epithelial‐mesenchymal transformation and affects tumor growth, angiogenesis, aggressiveness, and metastasis.^79^ Under the stimulation of PDGF signal transduction, cyclooxygenases (COX‐2) induces VEGF production in activated hematopoietic stem cells. TNP‐470 (a known cancer angiogenesis inhibitor) can inhibits the production of VEGF in PDGF‐activated hematopoietic stem cells, thus playing a role of antiangiogenesis.^80^ The novel indomethacin derivative, JI‐MT, has a potential prophylactic effect against HCC by inhibiting PDGFRα activation to terminate cell proliferation and angiogenesis.^81^ Another study showed that nuclear protein‐1 (NUPR1), a stress‐inducing protein overexpressed in various tumors, is upregulated by triiodothyroid hormone/thyroid hormone receptor (T3/TR) signaling. NUPR1 upregulates PDGFA expression, which motivates angiogenesis in HCC by activating the MEK/ERK signaling pathway. Therefore, the intervention of this angiogenic pathway may provide potential therapeutic targets for HCC (Figure 2).^82^

### 2.2 RAS/RAF/MEK/ERK pathway

The RAS/RAF/MEK/ERK signaling pathway is the main branch of the MAPK pathway.^83^ It takes part in many physiological processes, such as cell proliferation, apoptosis, invasion, metastasis, and regulation of inflammatory responses, and is closely related to the occurrence and progression of tumors.^84^, ^85^ RAS proteins with GTPase activity are activated by upstream RTK, and the activated RAS binds to the N‐terminal domain of RAF to activate RAF, which binds to downstream MEK proteins to activate MEK, and then further activated the phosphorylation of the downstream substrate ERK. Eventually, activated ERK gets into the nucleus, regulates different transcription factors expression and induces a sequence of physiological and biochemical reactions.^86^ Alterations of this pathway are widespread in tumors, and crosstalk between the RAS–RAF–MEK–ERK axis and other signaling pathways further amplifies its potential in cancer.^87^

The RAS/RAF/MEK/ERK pathway has a significant impact on HCC pathogenesis. Studies have found that 30% of HCC patients have mutations of RAS^88^ and RAF is overexpressed in most HCC tissues.^89^ Overexpression and overphosphorylation of MEK1/2 have been detected in more than 75% of HCC samples.^90^ Meanwhile, the MAPK/ERK signaling pathway can serve as a prognostic indicator for HCC patients, such as increased expression of the RAS effector is highly correlated with the low survival rate of patients with HCC.^91^

Inhibitors that target MEK are distinct from other kinase inhibitors because of their high specificity and lack of competition for adenosine triphosphate (ATP) binding. CI‐1040 was the first MEK inhibitor to be tested in clinical trials. Sorafenib in combination with CI‐1040 synergistically inhibits ERK phosphorylation and cell proliferation, and induces apoptosis in both HCC cells and human umbilical vascular endothelial cells (HUVEC). This combination therapy was significantly more effective in inhibiting HCC growth in animal models than either drug used alone.^92^ Most of the MEK inhibitors has shown to have insufficient antitumor activity and limited clinical efficacy.^93^ The MEK1/2 inhibitor selumetinib (AZD6244) has been shown to reduce ERK phosphorylation levels in HCC patients in phase II trials, but has low antitumor activity and poor efficacy in monotherapy.^94^ The second generation MEK inhibitor, PD 0325901, is about 500 times more potent than CI‐1040, and exhibits strong antitumor activity in many kinds of cancer.^95^ Salirasib is an S‐prenylcysteine analog that hampers liver cancer cell proliferation by inhibiting RAS/ERK expression and activity.^96^, ^97^ Trametinib is a selective inhibitor of MEK1 and MEK2, which has achieved clinical success, has be proved to improve the overall survival (OS) and progression‐free survival (PFS) of patients with BRAF V600E/K‐mutant melanoma.^98^ At present, several multitarget multikinase inhibitors related to RAS/RAF/MEK/ERK have been approved for market, but how to overcome the primary and acquired resistance is still a difficulty in the research and development of targeted drugs.

### 2.3 PI3K/AKT/mTOR pathway

The PI3K/AKT/mTOR pathway is hyperactive in almost all malignancies,^99^ making it an excellent therapeutic target. As a class of lipid kinases, PI3Ks are classified into three categories according to their substrate and structure specificity.^100^ On the basis of regulation mechanisms, Class I PI3K is further subdivided into subgroups IA and IB. Class IA PI3Ks are heterodimers comprising the p110 catalytic and p85 regulatory subunits.^101^ PI3K promotes the cytoplasmic transport of AKT1 to interact with phosphatidylinositol‐3,4,5‐diphosphate (PIP3) on the cell membrane, resulting in its activation through phosphorylation.^102^ phosphorylated AKT (p‐AKT) can suppress various downstream effectors, including Bcl‐2 agonist of cell death, Forkhead Box O1, and tuberous sclerosis complex (TSC1/2), to promote cell proliferation and survival.^103^

**FIGURE 3:**
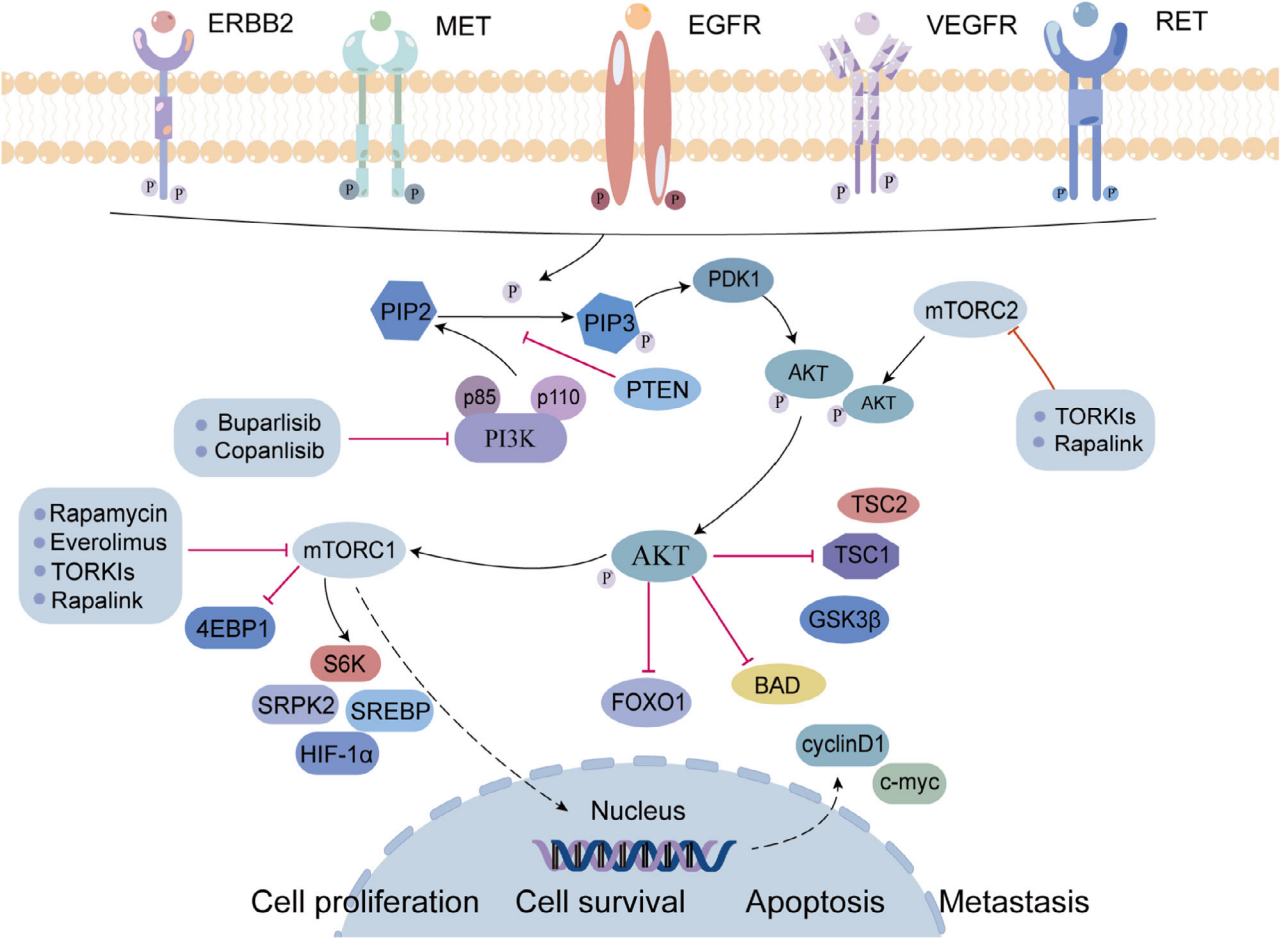
PI3K/AKT/mTOR signaling pathway and targeted inhibitors. Dysregulation of PI3K/AKT/mTOR signaling enhances the Warburg effect in tumors, promotes cell proliferation and metastasis in HCC. In addition, we describe the latest clinical trials of inhibitors targeting the PI3K/AKT/mTOR pathway. 4EBP1, eukaryotic translation initiation factor 4E‐binding protein 1; BAD, bcl‐2 agonist of cell death; ERBB2, human epidermal growth factor receptor‐2; FOXO1, Forkhead box O1; GSK‐3β, glycogen synthetase kinase‐3beta; MET, mesenchymal epithelial transfer factor; mTOR, mammalian target of rapamycin; PIP2, phosphatidylinositol‐4,5‐diphosphate; PIP3, phosphatidylinositol‐3,4,5‐diphosphate; PTEN, phosphatase and tensin homolog; PI3K, phosphoinositide 3‐kinase; RET, rearranged during transfection; S6K, S6 kinase; SREBP, sterol regulatory element binding protein; SRPK2, arginine protein kinase 2; TSC, tuberous sclerosis complex.

Overexpressed p‐AKT has also been considered to be capable of indicating poor prognosis in many malignant tumors.^104^ AKT is an important substrate of mTORC2, and mTORC1 is an important downstream target of AKT.^105^, ^106^ As a member of the PI3K‐related kinase family, mTOR participates in regulating cell growth and proliferation and perceiving nutritional signals.^107^ mTORC1 phosphorylates its downstream effector molecules, such as S6 kinase (S6K), eukaryotic translation initiation factor 4E‐binding protein 1 (4EBP1), sterol regulatory element binding protein (SREBP), to promote protein translation, nucleotide and lipid synthesis, lysosome biosynthesis, and to inhibit autophagy processes. mTORC2 involves in promoting cytoskeletal remodeling and cell migration, affecting metabolism and inhibiting apoptosis.^108^, ^109^ Phosphatase and tensin homolog (PTEN) is a dual phosphatase with both protein and lipid phosphatase activities. PTEN can dephosphorylate PIP3 to phosphatidylinostol‐4,5‐diphosphate (PIP2) to antagonizes PI3K. Since PTEN phosphatase activity inhibits PI3K, AKT1 can be negatively regulated. However, elevated PIP3 levels inhibit the above effects, leading to the constitutive activation of AKT and downstream cascades (such as mTOR signaling).^110^ Multiple mechanisms including mutations, methylation, loss of heterozygosity, protein instability, and abnormal expression of regulatory miRNAs can inactivate PTEN, and this phenomenon is common in human cancers (Figure 3).^110^, ^111^, ^112^

The PI3K/AKT/mTOR pathway is abnormally activated in nearly 50% of the patients with HCC.^113^ Targeting autophagy mediated by the PI3K/AKT/mTOR pathway can enhance drug sensitivity and prevent drug resistance in tumor cells.^114^ mTORC1 activates arginine protein kinase 2 (SRPK2, a regulator of RNA‐bound SR proteins) to promotes lipid biosynthesis, so it can serve as a potential therapeutic target for metabolic disorders.^115^ It has been shown that HIF‐2α is upregulated in hypoxic microenvironment and activates lipid synthesis via the PI3K/AKT/mTOR pathway, promoting the development of fatty HCC.^116^ In addition, the abnormal expression of 3‐phosphoinositide‐dependent protein kinase‐1 (PDK1), which is a key activator of the PI3K/AKT/mTOR signaling pathway, is characteristic of poorly differentiated invasive HCC cells, indicating that PDK1 can serve as a molecular target for HCC.^117^ Moreover, by inactivating PI3K/AKT/mTOR mediated autophagy, overexpressed suppressor of cytokine signaling 5 (SOCS5), a member of the suppressor of the cytokine signaling family, can promote the invasion and migration of HCC cells in vitro. Inhibiting both SOCS5 and mTOR has been found to be a potential treatment approach for restraining HCC metastasis and prolonging survival time of patients.^118^

According to their pharmacokinetic effects and interactions with ATP, PI3K inhibitors can be classified into three classes: pan‐PI3K, isotype‐specific PI3K, and dual PI3K/mTOR inhibitors.^119^ An ATP‐competitive pan‐PI3K inhibitor—buparlisib (BKM120)—targets all Class 1 PI3K.^120^ The results of a phase 3 clinical trial showed that buparlisib combined with endocrine therapy has clinical significance in postmenopausal women with advanced breast cancer resistant to endocrine therapy.^121^ And, Kirstein et al.^122^ found that buparlisib had anti‐HCC activity both in vitro and in vivo. It has been found that the most common buparlisib‐related adverse reactions (AEs) were rash, anorexia, hyperglycemia, nausea, mood changes, abnormal liver function, decreased appetite, and diarrhea.^123^ Copanlisib (BAY 80−6946) is also a pan‐PI3K inhibitor.^124^ Copanlisib was authorized by the United States Food and Drug Administration (US FDA) in 2017 for the therapy of relapsing follicular lymphoma, and it has been shown to strongly inhibit cell viability and colony formation.^125^ The first generation mTOR inhibitors are rapamycin and its analogs, which can specifically inhibit mTORC1.^126^ Studies have shown that rapamycin and its analogs inhibit the phosphorylation of mTOR both in vitro and in vivo, then effectively inhibiting HCC cell growth and proliferation.^127^ However, the inhibition of mTORC1 may cause feedback activation of IGF‐IR and AKT, which compromises the anticancer effects of rapamycin and its analogs.^128^ Second generation mTOR inhibitors (TORKIs) inhibit both mTORC1 and mTORC2, theoretically it is possible to prevent he phosphorylation of AKT by blocking mTORC2.^129^ However, second generation mTOR inhibitors are still associated with drug resistance. To solve the problem of therapeutic resistance in the first and second generation mTOR inhibitors, a third‐generation inhibitor, rapalink, was developed. There is evidence that rapalink can overcome the resistance of cancer cell to rapamycin, its analogs, and TORKIs.^130^ However, PI3K mono‐inhibitor drugs are prone to drug resistance, and it may be possible to improve the therapeutic effect by combining with other therapeutic drugs.

### 2.4 Wnt/β‐catenin pathway

The Wnt/β‐catenin signaling pathway, also known as the classical Wnt signaling pathway, is highly conserved during evolution and is involved in regulating tissue development and maintaining homeostasis in vivo. Abnormal Wnt/β‐catenin signaling contributes to the progression of cancers including cholangiocarcinoma (CCA) and HCC.^131^, ^132^

**FIGURE 4:**
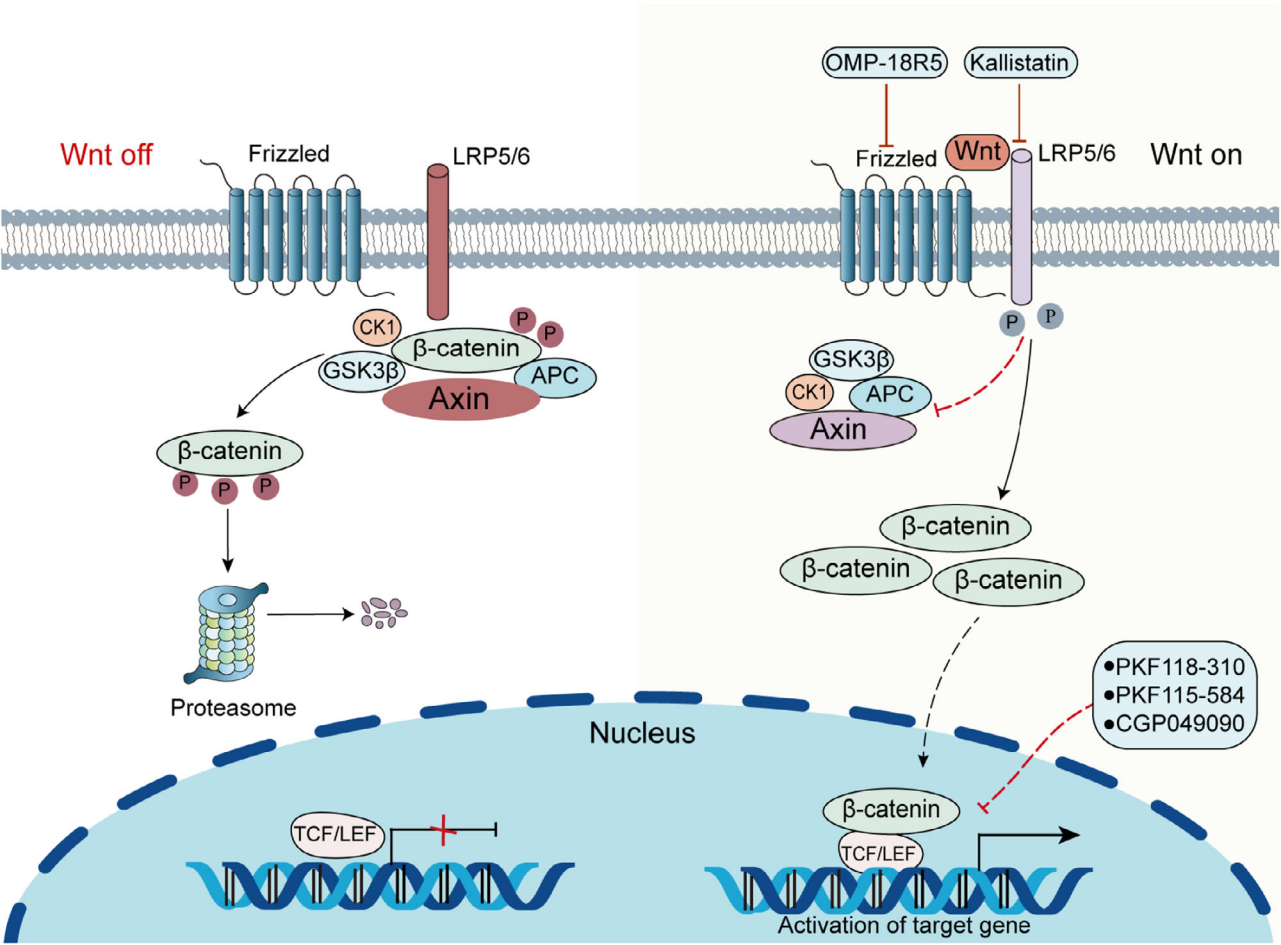
Wnt on and Wnt off state of Wnt/β‐catenin signaling pathway and therapeutic strategies based on them. The Wnt/β‐catenin pathway is normally inactivated in the mature healthy liver, being the Wnt off state. However, Wnt/β‐catenin signaling is frequently overactivated in HCC and in the Wnt on state. Molecular inhibitors and antibodies currently in clinical trials mainly target FZD, LRP5/6, β‐catenin, and TCF. APC, adenomatous polyposis coli; Axin, scaffold protein axin; CK1, casein kinase 1; FZD, Frizzled; LRP5/6: lipoprotein reception‐associated protein; LEF, lymphoid enhancer‐binding protein family; TCF, T‐cell factor.

Wnt is a secreted glycoprotein that interacts with the signaling‐receiving Frizzled (FZD) protein on the cell membrane to activate downstream signaling pathways.^133^ FZD receptors are seven‐pass transmembrane proteins and are the primary receptors of the Wnt pathway.^134^ The Wnt protein can bind to the FZD protein and a single transmembrane lipoprotein reception‐associated protein (LRP5/6) to form the Wnt/FZD/LRP complex in the presence of Wnt ligand,^135^ and then activates the signaling pathway, causing an increase in cytoplasmic β‐catenin concentration. β‐Catenin is deposited to a certain extent and enters the nucleus, where it binds to T cell factor (TCF) and lymphoid enhancer‐binding protein family (LEF) transcription factors to co‐regulates transcription of downstream genes responsible for proliferation and cell survival.^136^, ^137^ The degradation of β‐catenin is regulated by the β‐catenin destruction complex, which comprises the adenomatous polyposis coli (APC), scaffold protein axin (Axin), and two kinases, casein kinase 1 (CK1) and glycogen synthetase kinase‐3beta (GSK‐3β).^138^, ^139^ However, when Wnt binds to FZD and LRP, the destruction complex is inactivated (Figure 4).^140^

β‐catenin is a kind of multifunctional protein encoded by the human CTNNB1 gene.^141^ CTNNB1 mutation is the main oncogenic change in liver cancer with the mutation rate of 20−40%. CTNNB1 mutations lead to the increased stability of β‐catenin, thus, the target gene is upregulated.^142^ Researches have pointed out that the activation of the Wnt/β‐catenin pathway can restore tissue integrity after acute liver injury. However, persistent abnormal activation often leads to cancer.^143^, ^144^ In early HCC, β‐catenin is mainly localized in the cell membrane and forms a complex with multiple cadherin family members to promote tumor cell survival by enhancing the signaling of growth factor receptors, such as EGFR.^145^ Matrix metalloproteinases (MMPs) are involved in increasing the migration and invasion of cancer cells, while the activation of Wnt signaling and nuclear translocation of β‐catenin can enhance the expression of MMPs and promote the migration of HCC.^146^

Drugs targeting the Wnt/β‐catenin pathway can be divided into two main subtypes: monoclonal antibodies (mAbs) and small molecule inhibitors (SMIs). SMIs can bind to receptors with a stronger affinity than the original ligands, and monoclonal antibodies not only act directly on extracellular and membrane‐bound regions, but also activate the innate internal immune system to exert antitumor effects indirectly.^147^ Small‐molecule inhibitors (PKF118‐310, CGP049090, and PKF115‐584) that block the interaction between β‐catenin and TCF have been found to induce apoptosis and inhibit the growth of HCC cells.^148^ Anti‐FZD monoclonal antibodies such as OMP‐18R5 inhibit the growth of various tumor types.^149^ Another study suggested that kallistatin plays an antiangiogenic role by inhibiting Wnt signaling by antagonizing LRP6.^150^ It has also been reported that N‐myc expression can be inhibited in HCC cells and tissues by acyclic retinoid (ACR), a synthetic vitamin A derivative, thereby the Wnt/β‐catenin signaling pathway is blocked and tumor growth is slowed down.^151^ In HCC cells, cytochrome P450 2E1 (CYP2E1) overexpression significantly inhibits the activity of β‐catenin signaling pathway, thereby inhibiting the development HCC.^152^ It is worth noting that it is important to further develop Wnt/β‐catenin signaling targeted drugs and improve the specificity and safety of targeted drugs in HCC.

### 2.5 JAK–STAT pathway

**FIGURE 5:**
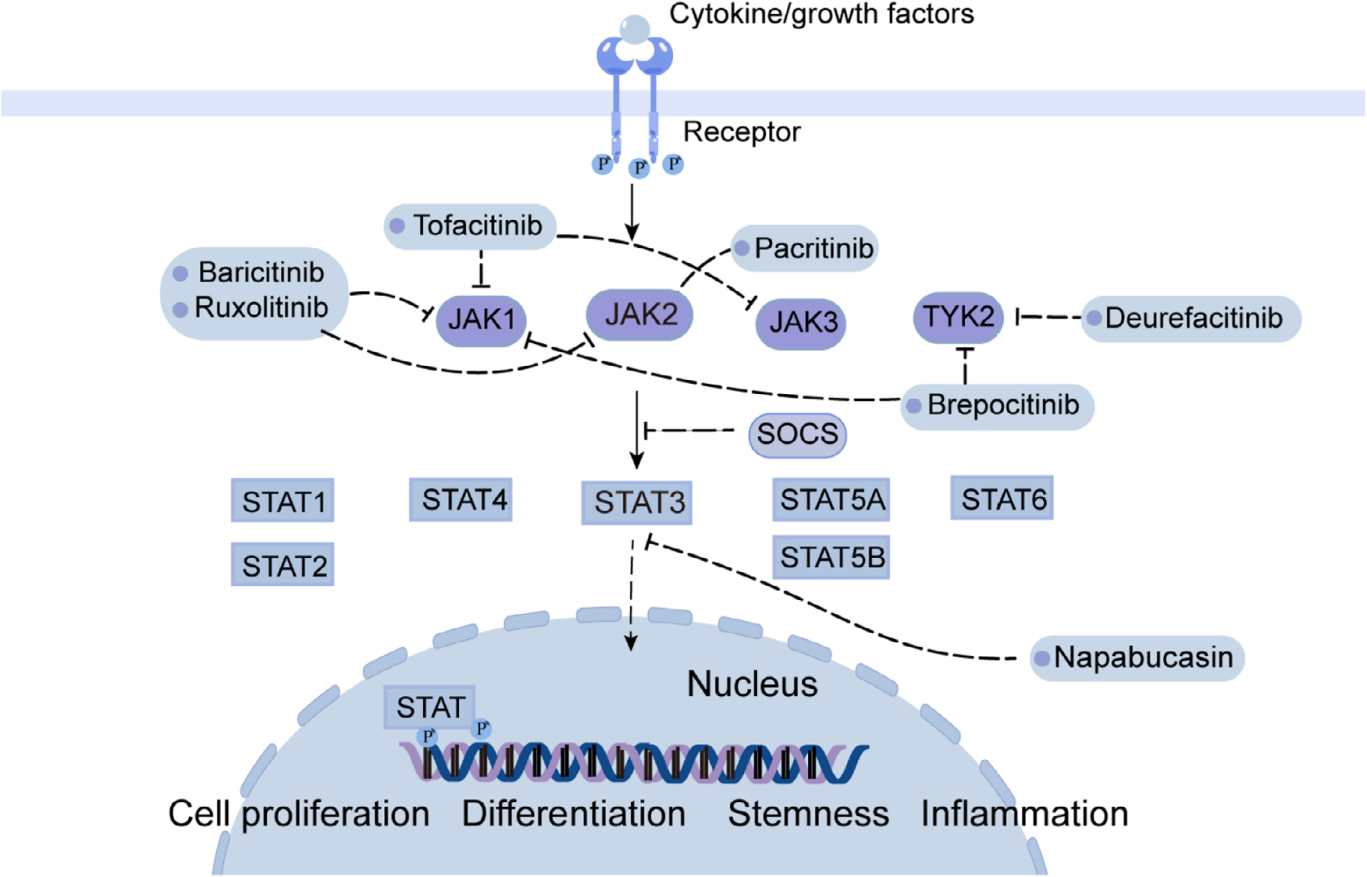
Schematic of JAK/STAT signaling and targeted inhibitors. The JAK/STAT signaling pathways regulate gene expression and cell physiological functions such as cell proliferation, differentiation, cell metabolism, and immune response. Currently developed inhibitors mainly target JAK1‐3, TYK2, and STAT3. JAK, Janus‐kinase; STAT, signal transducer activator of transcription; SOCS, suppressor of cytokine signaling; TYK2, tyrosine kinase 2.

The JAK–STAT pathway is highly conserved and comprises JAK1‐3, TYK2, and a class of downstream JAK target proteins (STAT1‐6). It participates in the regulation of cell proliferation, stem cell maintenance and differentiation, and immune/inflammatory response.^153^, ^154^ Oxidative stress, epigenetic silencing of SOCS genes, growth factor stimulation, and increased inflammatory signaling contribute to the activation of JAK/STAT signaling pathway.^155^ JAKs receptors bind to cytokines and then JAKs pathway can be activated. Activated JAKs initiate phosphorylation and recruit the corresponding STATs . Subsequently, activated STAT homologous dimers migrate to the nucleus and activate target genes like recombinant Cyclin D1 (CCND1), myeloid cell leukemia‐1 (Mcl‐1), and Survivin.^156^, ^157^ JAK/STAT signaling is negatively regulated by SOCS proteins, which are induced by activated STAT dimerization that enters the nucleus. Binding of SOCS protein to the phosphorylated JAK and its receptors results in the inhibition of JAK/STAT signaling (Figure 5).^158^

JAK2 phosphorylates histone (H3), consequently reducing the affinity between H3 and HP1α and promoting tumorigenicity.^159^ STAT3 and STAT5A/B can promote cancer development, whereas STAT1 is a cancer suppressor.^160^, ^161^ In patients with HCC, STAT1 is mainly present in the form of unphosphorylated‐STAT1, while phosphorylated‐STAT1 has an antitumor effect.^162^ Inhibition of STAT1 activity in patients with HCC is correlated with VEGF levels, suggesting that the antitumor effects of STAT1 is caused by inhibiting angiogenesis.^163^ STAT2 plays an antitumor role in liver cancer by inhibiting oncogene transcription.^164^ Studies have shown that STAT3 is the most highly expressed JAK/STAT pathway protein in HCC, and high expression of STAT3 indicates poor prognosis of HCC, therefore, STAT3 is considered as a true oncogene that promotes HCC development.^165^, ^166^ STAT3 is strongly associated with malignancies and plays key roles in tumor cells, stromal cells, and tumor‐resident immune cells that regulate cancer development.^167^ STAT3 can promote the expression of antiapoptotic genes such as BIRC5, BCL2L1/Bcl‐xL (BCL2‐like 1), and Mcl‐1.^168^, ^169^, ^170^ The STAT3 signaling pathway protects cells from different apoptotic stimuli. In addition, sorafenib accelerates STAT3 dephosphorylation and make CCA cells sensitive to apoptosis.^171^, ^172^, ^173^, ^174^

Drugs targeting the JAK/STAT pathway can be divided into three categories: (1) cytokine or receptor antibodies, (2) STAT inhibitors, and (3) JAK inhibitors. Jakinibs, which are first generation JAK inhibitors, include tofacitinib, baricitinib, and ruxolitinib. Tofacitinib inhibits all JAK and is used to treat autoimmune diseases and organ transplant rejection.^175^ Baricitinib inhibits both JAK1 and JAK2 and can be used to treat patients with rheumatoid arthritis.^176^ Another small molecule inhibitor of JAK1 and JAK2: ruxolitinib, inhibits HCCLM3 cell line metastasis both in vivo and vitro.^177^ In 2019, upadacitinib, a relatively selective JAK1 inhibitor, was authorized to the treatment of RA.^178^ Pacritinib, another effective and selective JAK2 inhibitor, reduces liver fibrosis in mouse models, hampering the development of hepatic steatosis and progression of clinical HCC.^179^ Jakinib is also effective in patients of severe autoimmune and immune disorders with STAT1 or STAT3 mutations.^180^ Similar adverse effects can be found in all JAK inhibitors, including hyperlipidemia, infection, and cytopenia.^181^

Napabucasin, a STAT3 inhibitor, stimulated apoptosis in vitro, inhibited HCC growth in in‐situ mouse models, and reduced the recurrence of HCC after hepatectomy.^182^ Tyrosine kinase 2 (TYK2) inhibitor deurefacitinib is the first drug to target the kinase‐like domain, and the TYK2/JAK1 inhibitor brepocitinib is the other selective inhibitor.^183^ The blockade of JAK–STAT signaling can as well inhibit tumor surveillance of immune cells, which may reduce the efficacy of jakinib in solid tumors.^184^

### 2.6 Hh pathway

The Hh signaling pathway comprises Hh ligands (Indian Hedgehog—Ihh, Desert Hedgehog—Dhh, and Sonic Hedgehog—Shh), Patched‐1/2 (Ptch‐1), Smoothened (Smo), glioma related oncogene homology transcription factors −1/2/3 (Gli‐1/2/3), kinesin family member protein 7 (Kif7), and protein kinase A (PKA).^185^ Hh is a classical morphogenetic factor that regulates embryonic development and tissue repair, and contributes to angiogenesis, tumorigenesis, and metastatic seeding. When Hh ligand is present in extracellular environment, it binds to Ptch‐1 on the cell membrane, and then Ptch‐1 is endocytosed, abolishing Smo inhibition and allowing Smo to accumulate and send a signal to the suppressor of fused homolog (Sufu) to release Gli activator (GliA). Then GliA migrates to the nucleus and activates target gene expression.^186^

**FIGURE 6:**
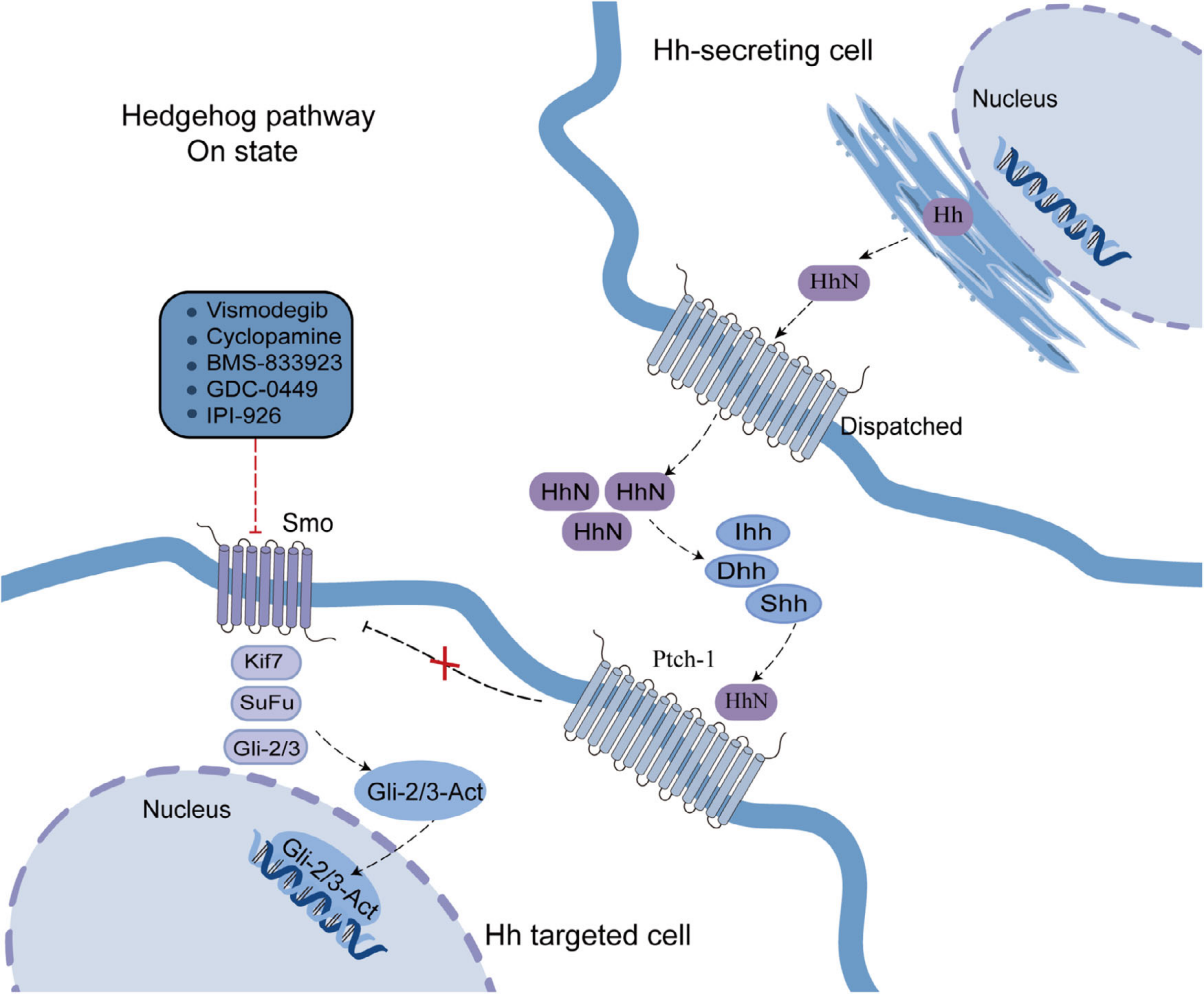
Schematic diagram of Smo‐driven canonical Hh signaling pathway and targeted inhibitors. Hh signaling is controlled by the Ptch and Smo. In the absence of Hh and Ptch, activation of Smo leads to the activation of Hh target genes. Dhh, Desert Hedgehog; Gli, glioma related oncogene homology transcription factors; Hh, hedgehog; HhN, amino‐terminal “Hedge” domain; Ihh, Indian Hedgehog; Kif7, kinesin family member protein 7; Ptch, Patched; Smo, Smoothened; Shh, Sonic Hedgehog; SuFu, suppressor of fused homolog.

Under normal conditions, the adult Hh signaling pathway is almost silent in tissues, and abnormal activation of Hh signaling pathway can lead to cancer. Mutation or amplification of critical ingredients of the Hh signaling pathway (such as Ptch functional mutation loss of Smo inhibition or functional mutation of Smo activation) induces abnormal constitutive activation of this pathway, leading to tumors.^187^, ^188^ Hh ligand‐dependent mechanisms include autocrine and paracrine signaling. Tumor cells secrete Hh ligands, bind to Ptch, and abnormally activate Hh signaling. Simultaneously, along with paracrine signals, the Ptch1 receptor in tumor stromal cells binds to the Hh ligand and activates the Hh pathway, which further transmits growth signals (VEGF, IGF, Wnt, and PDGF) to tumor cells in a feedback loop, promoting the proliferation and differentiation of tumor cells (Figure 6).^189^

Hh signal activation occurs frequently in HCC, and it has been found that over 60% of HCC tissues have high expression of Shh, Ptch1, and Gli1.^190^ HCC Patients with tumor metastasis, chemotherapy resistance, poor prognosis, low overall survival, and recurrence after liver transplantation are often accompanied by overexpression of the Hh signaling pathway.^191^ Hh pathway components are overexpressed and Hh downstream transcription factors are activated in HCC cell lines HepG2 and Hep3B. In addition, it has been confirmed that Hh signaling inhibits apoptosis and promotes proliferation, invasion, and migration of HCC cells.^192^ The Smo blocker KAAD‐cyclopamine inhibited Hh signaling, reduced the expression of the HCC oncogene c‐myc, and inhibited the growth of Hep3B cells.^193^

Aberrant activation of Hh signaling can also be observed in different animal models of HCC, such as multidrug resistance protein‐2‐deficient mice fed with chronic alcohol, idiopathic fibrotic bile duct lesions, and xenotransplantation models of primary liver cancer.^194^ In mice, gene‐induced Hh activation in 2–5% of liver cells enhances oncogene‐induced HCC.^195^ Smo inhibitors have been reported to reduce tumor size, inhibit angiogenesis and metastasis,^196^, ^197^ and increase radiosensitivity in vivo.^198^ A phase I clinical trials (ClinicalTrials.gov: NCT0215864) about Smo inhibitor is ongoing.^199^ Smo inhibitors have been successfully used against other solid cancers, such as basal cell carcinoma.

### 2.7 Hippo pathway

The Hippo signaling pathway includes a group of conserved kinases. In mammals, Hippo pathway is composed of mammalian sterile 20‐like kinase1/2 (MST1/2), large tumor suppressor kinase 1/2 (LATS1 and LATS2), Salvador homologous protein 1 (SAV1), MOBKL1A/B (MOB1A/B), transcription regulatory factors with WW domains (TAZ), yes associated protein 1 (YAP), and transcriptional enhanced associate domain 1 (TEAD1). This pathway participates in the regulation of tissue development, maintenance of tissue homeostasis, regenerative repair by regulating stem cell self‐renewal, cell proliferation, survival, differentiation, and apoptosis.^200^ The phosphorylation of LATS1/2 and MOB1A/B as well as the inhibition of YAP and TAZ activity can occur when the Hippo pathway is activated. Dephosphorylated YAP/TAZ can enter the nucleus when the Hippo pathway is shut down and bind to the transcription factors TEAD1‐4 to further promote gene expression.^201^ Aberrant activation of YAP/TAZ and TEAD1‐4 leads to activation of other components of the pathway, leading to metastatic progression of tumors. Thus, disrupting the interaction of YAP/TAZ and TEAD1‐4 can inhibit cancer progression.

The dysregulation of the Hippo pathway is vital to the development of HCC. Genetic disruption of Hippo signaling molecules, such as NF2, WW45, and YAP, leads to persistent liver overgrowth and HCC development.^202^, ^203^ Zhao et al.^204^ used immunohistochemical staining to assess YAP expression in HCC tissues, and they discovered that 95% of normal liver tissues were amblychromatic, but 63 (54%) of 115 HCC samples showed strong YAP staining, indicating a considerable difference in YAP protein levels between normal and HCC tissues. Through stimulating the multiplication of hepatocytes in a mouse model with a liver‐specific transgenic mutation, YAP plays a significant part in the development of HCC.^205^ Experiments have shown that inducing YAP overexpression can increase the apoptosis resistance of doxorubicin‐induced HCC cells, while interfering with YAP expression reverses YAP‐enhanced chemoresistance.^206^ Activation of YAP plays an important part in the development of HCC, and it is possible that deregulation of the Hippo pathway is a common mechanism for YAP activation.

Verteporfin, an US FDA‐approved YAP inhibitor, binds to YAP and disrupts its action in TEAD.^207^ Verteporfin can reduce the mRNAs expression of Hippo pathway target genes, such as cysteine‐rich angiogenic inducer61 (CYR61), connective tissue growth factor, AXL, as well as BIRC‐5.^208^ The injection of verteporfin in YAP transgenic mice resulted in a reduction in liver size, whereas in wild‐type mice, it had no effect on liver tissue, suggesting its potential application as a therapeutic target in patients diagnosed with HCC.^209^

## 3 TARGETED THERAPY IN HCC

Targeted therapy in HCC has been developing in the past decade. Sorafenib is the main first‐line therapy for HCC. Subsequently, lenvatinib, bevacizumab (BVZ), and donafenib provide more options for the first‐line therapy of HCC. In 2017, the OS of HCC patients who had progressed after previous treatment with sorafenib was significantly improved after taking regorafenib, indicating the arrival of second‐line targeted therapies. The subsequent emergence of ramucirumab and cabozantinib has brought new options for HCC treatment.

### 3.1 First‐line targeted therapies

#### 3.1.1 Sorafenib

Sorafenib is a commonly used clinical multitarget tyrosine kinase receptor inhibitor, it can inhibit VEGFR, PDGFR‐β, and RAF/MEK/ERK signaling pathways, thereby inhibiting tumor cell proliferation and blocking tumor neovascularization.^210^ To our knowledge, sorafenib is the first systematic drug for the treatment of advanced HCC. In a multicenter, double‐blind, placebo‐controlled phase III trial (ClinicalTrials.gov: NCT00105443), 602 patients with advanced HCC who had not previously undergone systemic therapy were chosen at random to receive sorafenib or placebo treatment. In the sorafenib group (with 400 mg of sorafenib twice daily), the median overall survival (mOS) was 10.7 months, while it was 7.9 months in the placebo groups. The median time to symptom progression (mTTP) between the two groups was 4.1 m versus 4.9 m without significantly different. Hand and foot skin reactions (10.7%), diarrhea (6.0), and fatigue (3.4%) were mainly grade 3/4 drug‐related side effects in the sorafenib group.^211^ The treatment efficacy of sorafenib was also supported by a follow‐up randomized controlled trial in the Asia‐Pacific region (ClinicalTrials.gov: NCT00492752): A randomized, double‐blind, placebo‐controlled phase III trial evaluated the efficacy and safety of sorafenib in HCC patients who had not received systemic therapy and had Chid‐Pugh liver function class A. mOS was 6.5 months in the sorafenib group (*n* = 150), 4.2 months in the control group (*n* = 76), and mTTP was 2.8 months for sorafenib versus 1.4 months for placebo. The most common grade 3/4 drug‐related side effects were hand and foot skin reactions (8%) and diarrhea (8%).^212^ However, the subjects in these two trials had good liver function (compensatory cirrhosis), and the degree of cirrhosis affected HCC prognosis.

The GIDEON trial (ClinicalTrials.gov: NCT00812175) aimed to validate the therapeutic effects of sorafenib in patients with unresectable HCC (including Child‐Pugh class B). The majority of patients, regardless of Child Pugh status, took the recommended 800 mg sorafenib initial dose. The general safety profile and dosing approach are comparable among Child‐Pugh subgroups.^213^ It showed that within the intent‐to‐treat population (*n* = 3213), the mOS for Child‐Pugh A patients was greater (13.6 months) than for Child‐Pugh B patients (5.2 months), while the mTTP was comparable (4.7 vs. 4.4 months, respectively).^214^ The US FDA defines sorafenib as the first line treatment for inoperable or advanced HCC. Sorafenib can also be used as antitumor therapy in patients who relapse after liver transplantation. Side effects of sorafenib include diarrhea, nausea, vomiting, skin reactions on the hands and feet, fatigue, and hypertension. In addition, basic liver function, clinicopathology, and etiology affect the prognosis of patients with sorafenib treatment. The success of clinical trials described above, promoted the approval of sorafenib as first‐line targeted therapy for advanced HCC.^215^ Sorafenib has been the first‐line therapy for decades, while new treatment options are being applied in clinical practice with the progress of research.

#### 3.1.2 Lenvatinib

Lenvatinib, a multitarget TKI, has demonstrated excellent efficacy in the management of a number of solid tumors. Its main targets include VEGFR, FGFR, PDGFR, RET, and KIT. They also have immunomodulatory effects. Lenvatinib inhibits angiogenesis by fully blocking VEGFR signaling pathway, leading to tumor “starvation” and hypoxia, resulting in growth inhibition or death. It also modulates tumor immune microenvironment by inhibiting VEGF pathway. Simultaneously, it can inhibit the PDGF/PDGFR/KIT/RET and FGFR pathways, resulting in stronger antitumor activity. In addition, by blocking FGFR‐4, lenvatinib decreases PD‐L1 expression in cancer cells and inhibits Treg differentiation to play an immunomodulatory role, demonstrating a positive interaction with immunotherapy.^216^

In 2018, the US FDA and China's NMPA successively approved lenvatinib as a first line therapeutic option for unresectable HCC patients. In a randomized, global, multicenter, noninferiority phase III clinical study (ClinicalTrials.gov: NCT01761266), patients were randomly divided into two groups, taking lenvatinib (*n* = 478) and sorafenib (*n* = 476), respectively, and the results showed that the OS rate of the lenvatinib group was noninferior. The OS tended to be longer than that of sorafenib group (13.6 months vs. 12.3 months). In the lenvatinib group, the median progression‐free survival (mPFS) was 7.4 months, the median progression‐stage mTTP was 8.9 months, and the ORR was 24%. The mPFS and mTTP of the sorafenib group were both 3.7 months, and the ORR was 9%. The statistical results showed that the treatment response in the lenvatinib group was significantly improved compared with the sorafenib group in terms of secondary efficacy endpoints. As for drug safety, there was no discernible difference between the lenvatinib and sorafenib groups. The incidence of treatment‐related adverse events (TRAEs) in the lenvatinib and sorafenib groups was similar, with 13 and 9% of patients discontinuing the treatment due to TRAEs, respectively.^217^ Based on these results, lenvatinib can be regarded as an alternative first‐line treatment option for patients with advanced HCC. The approval of lenvatinib further consolidated the role of multikinase inhibitors in the first‐line treatment of advanced HCC.

#### 3.1.3 Bevacizumab

BVZ is a recombinant IgG1 monoclonal antibody with high affinity to VEGF and prevents its interaction with VEGFR, thereby inhibiting signal pathway transduction.^218^ BVZ inhibits VEGFR activation, stimulates tumor cell apoptosis, and inhibits pro‐angiogenesis signaling pathways. Animal models used in preclinical studies have demonstrated that BVZ treatment inhibits vascular growth, reduces blood supply, and decreases vascular permeability within tumors, thereby inhibiting tumor progression.^219^ BVZ also enhances the effect of chemotherapy by inhibiting tumor angiogenesis and reducing the increase of tumor tissue pressure. A phase II study (ClinicalTrials.gov: NCT00055692) showed that BVZ had significant clinical and biological effects in patients with nonmetastatic HCC, with an ORR of 13% and a mPFS of 6.9 months, and mainly grade 3−4 adverse events were hypertension (15%) and thrombosis (6%).^220^ A phase III clinical trial, IMbrave150 (ClinicalTrials.gov: NCT03434379), indicated that individuals with unresectable HCC who had not previously undergone systemic treatment reacted considerably better with atezolizumab + BVZ compared with sorafenib in terms of OS and PFS outcomes. The PFS of the atezolizumab + BVZ group was 6.8 months compared with 4.3 months for the sorafenib group. In comparison with sorafenib, atezolizumab + BVZ group was linked to 42% decreased risk of death and noticeably longer OS. Hypertension, albuminuria, and bleeding are the primary side effects of BVZ, while minor intestinal bleeding is rare.^221^ Atezolizumab in combination with BVZ has become the current standard of care for first line treatment of unresectable or metastatic HCC.^222^

#### 3.1.4 Donafenib

Independently developed in China, donafenib is a molecular‐targeted drug produced by the chemical modification of the molecular structure of sorafenib. It can simultaneously inhibit the activity of RTKs such as VEGFR and PDGFR. It can also directly inhibit various RAF kinases and downstream RAF/MEK/ERK signal pathways, inhibit the proliferation of tumor cells and the formation of tumor blood vessels, and play an antitumor role in dual inhibition and multitarget blocking.^223^

In a phase II/III research (ClinicalTrials.gov: NCT02645981), donafenib was evaluated as a first line treatment for advanced HCC in 668 HCC patients from March 2016 to April 2018. At the 56th Annual Meeting of the American Society of Oncology (ASCO2020), researchers presented the latest research results worldwide, demonstrating that donafenib has made a breakthrough in targeted therapy for HCC. Patients were randomized 1:1 to donafenib (200 mg twice daily) or sorafenib (400 mg twice daily). The outcomes demonstrated that the OS was superior in the donafenib group compared with the sorafenib control group. The mOS in donafenib group was 12.1 months as compared with 10.3 months in the sorafenib group, representing a 17% reduction in risk in the donafenib group. In terms of safety, AEs were basically similar between the two groups. Common AEs in the donafenib group included hand and foot skin reactions (50.5%), diarrhea (36.6%), elevated aspartate aminotransferase (AST) levels (40.5%), elevated blood bilirubin levels (39.0%), and thrombocytopenia (37.8%). The number of serious AEs and adverse events leading to the reduction or suspension of medication in the donafenib group was significantly lower, and the toxic side effects were also lower in the donafenib group compared with the sorafenib group.^224^ In June 2021, donafenib was officially approved for marketing in China as a first line therapy for advanced HCC patients with inoperable or distant metastases.

Nevertheless, resistance to first‐line targeted agents reduces their beneficial effects, second‐line therapies are urgently needed.

### 3.2 Second‐line targeted therapies

#### 3.2.1 Regorafenib

Regorafenib is a multitarget kinase inhibitor that resembles sorafenib structurally. Regorafenib inhibits VEGFR, FGFR‐1, PDGFR, KIT, RET, and B‐RAF, hampering tumor growth by inhibiting angiogenesis and maintaining the TME. It obtained US FDA approval in April 2017 to treat HCC patients who were no longer responsive to sorafenib. Regorafenib was approved by the China Food and Drug Administration in December 2017 for HCC patients who have previously taken sorafenib therapy but are well tolerated. Regorafenib approval was based on RESORCE, a multicenter, randomized, international, double‐blind, placebo‐controlled phase III clinical trial (ClinicalTrials.gov: NCT0177434) that enrolled 573 HCC patients with Barcelona Clinic Liver Cancer stage B or C who were resistant to sorafenib therapy. The mOS of the regorafenib group was 10.6 months, and the mOS of the placebo group was 7.8 months, indicating that the OS of HCC patients receiving regorafenib was significantly improved. Meanwhile, PFS was significantly extended based on the RECIST criteria modified for liver cancer, with mPFS of 3.1 months in the regorafenib group and 1.5 months in the placebo group. Based on the modified RECIST, the ORR were 11 and 4% in the regorafenib and placebo group, respectively.^225^ Regorafenib is the first systemic therapy drug to show survival benefit in patients with HCC who progressed on sorafenib.

#### 3.2.2 Cabozantinib

Cabozantinib is a multitarget small molecule TKI that can inhibit MET, VEGFR, ROS1, RET, AXL, NTRK, KIT, and so on.^226^ Cabozantinib was approved by the US FDA for second line treatment of advanced HCC in January 2019. 707 HCC patients with Child‐Pugh Class A who were resistant to previous sorafenib treatment and had disease progression after at least one systematic treatment were included in the double‐blind phase III trial (ClinicalTrials.gov: CT01908426). They receive either cabozantinib (60 mg once daily) or placebo with random assignment in a 2:1 ratio. The study results revealed that the cabozantinib group had considerably longer mOS (10.2 months vs. 8.0 months) and mPFS (5.2 months vs. 1.9 months) than the placebo group, with ORRs of 4% and less than 1%, respectively. 68% of patients in the cabozantinib group experienced grade 3 or 4 side effects, compared with 36% in the placebo group. Hand‐foot skin reactions (17%), hypertension (16%), elevated AST levels (12%), fatigue (10%), and diarrhea (10%) were common side effects in the cabozantinib group.^227^

#### 3.2.3 Ramucirumab

Ramucirumab is an IgG1 monoclonal antibody that binds to VEGFR‐2, which can efficiently prevent VEGFR‐2 binding to VEGF‐A, C, and D, thereby inhibiting downstream signaling pathways and affecting physiological activities, such as angiogenesis.^228^ Ramucirumab inhibits VEGFR‐2 phosphorylation mediated by VEGF and then inhibits activation of its downstream signaling molecules (phosphoinositide phospholipase C‐γ, MAPK), hampering the migration and proliferation of tumor cells.^229^ In phase I clinical trials (Clinicaltrials.gov: NCT00786383), ramucirumab demonstrated antiangiogenesis (reduction of tumor perfusion and vessel density) and antitumor activities in advanced cancer patients.^230^ In a phase Ib/II study (ClinicalTrials.gov: NCT02082210), the combination of emibetuzumab and ramucirumab was safe and tolerable in advanced HCC patients.^231^ A phase III trial (ClinicalTrials.gov: NCT02435433), which studied 565 patients with advanced HCC treated with ramucirumab monotherapy after first line sorafenib treatment, demonstrated no discernible improvement in OS against placebo group (*p* = 0.14). However, ramucirumab significantly improved mOS (8.5 months vs. 7.3 months, *p* = 0.0199) and mPFS (2.8 months vs. 1.6 months, *p* < 0.0001) compared with placebo group in a subset of patients with an α‐fetoprotein (AFP) concentration ≥400 ng/mL (*n* = 250); the ORR was 4.6 and 1.1%, respectively (*p* = 0.1156). The treatment was well tolerated, and adverse events of grade 3 or above were mainly hypertension (12.2%) and hyponatremia (5.6%). Ramucirumab has been shown to be effective and safe in sorafenib‐tolerant and advanced HCC patients with high AFP level.^232^ US FDA approved ramucirumab for advanced HCC with AFP level ≥400 ng/mL in May 2019.

#### 3.2.4 Apatinib

Apatinib is a TKI that selectively inhibits VEGFR‐2, thereby blocking the intracellular VEGF signaling pathway.^233^ Apatinib significantly inhibited the invasion, migration, and angiogenesis of Hep3B cells by blocking the VEGF, PI3K/AKT pathways.^234^ Apatinib inhibits the phosphorylation of VEGFR‐2 in HUVECs and prevents tube formation and endothelial cell migration in vitro.^235^ A study evaluating the efficacy of apatinib and sorafenib in HCC using multimodal molecular imaging showed that apatinib has antitumor and antiangiogenic effects comparable to sorafenib, and has fewer toxic effect.^236^ 23 advanced HCC patients were enrolled between December 2016 and June 2018 in a single‐arm, open‐label phase II research (ClinicalTrials.gov: NCT03046979) to assess the effectiveness, safety, and toxicity of apatinib. The results showed that apatinib had great clinical efficacy, safety, and tolerance in advanced HCC patients, and its toxicity was within the acceptable range. The ORR was 30.4%, while the disease control rate (DCR) was 65.2% in the apatinib group. The mOS and PFS were 13.8 and 8.7 months, respectively. The common TRAEs were albuminuria (39.1%), hypertension (34.8%), and hand and foot skin reactions (34.8%).^237^ In a phase III trial (ClinicalTrials.gov: NCT02329860), 400 eligible patients with advanced HCC were randomly assigned to receive apatinib (*n* = 267) or placebo (*n* = 133). The results showed that OS was improved in apatinib group compared with the placebo group, with the mOS 8.7 months versus 6.8 months and mPFS 4.5 months versus 1.9 months, respectively. The most common treatment‐related grade 3 or 4 adverse events were hypertension (28%), hand and foot skin reactions (18%), as well as decrease platelet count (13%). This indicates that apatinib significantly decreased the risk of death in HCC patients who had previously been resistant to chemotherapy or targeted therapy. Consequently, apatinib was approved to be used as a treatment in the second line for patients who had advanced HCC on December 31, 2020 (Table 1).^238^

**TABLE 1:** Clinical application of first‐ and second‐line targeted drugs for the treatment of advance hepatocellular carcinoma since 2008.

Targeted drugs	Targets	Trial design	NCT number	Primary endpoint	Grade 3/4 drug‐related side effects (%, trial drug)	Indication	References
First‐line treatment							
Sorafenib	VEGFR, PDGFR‐β, RAF	Phase III RTC (sorafenib vs. Placebo)	NCT00105443	mOS 10.7 vs. 7.9 months	Hand‐foot skin reactions (10.7), diarrhea (6.0), fatigue (3.4)	First‐line therapy for advanced HCC patients with inoperable or distant metastases	[211]
		Phase III RTC (sorafenib vs. Placebo)	NCT00492752	mOS 6.5 vs. 4.2 months	Hand‐foot skin reactions (8), diarrhea (8), weight loss (2), hypertension (2)		[212]
Lenvatinib	VEGFR, FGFR, PDGFR, RET, KIT	Phase III RTC (Lenvatinib Vs. sorafenib)	NCT01761266	mOS 13.6 vs. 12.3 months	Hypertension (42), diarrhea (39), decreased appetite (39), decreased weight (31)	First‐line therapy for advanced HCC patients with inoperable or distant metastases	[217]
**Bevacizumab**	VEGF‐A	Phase III RTC (atezolizumab and bevacizumab vs. sorafenib)	NCT03434379	mOS > 17 vs. 13.2 months	Hypertension (15.2), fatigue (2.4), increased AST (7), increased ALT (3.6)	First‐line therapy for advanced HCC patients with inoperable or distant metastases	[221]
Donafenib	VEGFR, PDGFR	Phase II/III RTC (donafenib vs. sorafenib)	NCT02645981	mOS 12.1 vs. 10.3 months	Hypertension (9), hand‐foot skin reactions (6), diarrhea (2), decreased platelet count (4)	First‐line therapy for advanced HCC patients with inoperable or distant metastases	[224]
Second‐line treatment							
Regorafenib	VEGFR, FGFR, PDGFR, KIT, RET, B‐RAF	Phase III RTC (regorafenib vs. Placebo)	NCT0177434	mOS 10.6 vs. 7.8 months	Hypertension (15), hand‐foot skin reactions (13), fatigue (9), diarrhea (3)	Advanced HCC patients who have failed prior first‐line therapy	[225]
Cabozantinib	MET, VEGFR, ROS1, RET, AXL, NTRK, KIT	Phase III RTC (cabozantinib vs. placebo)	NCT01908426	mOS 10.2 vs. 8.0 months	Hypertension (16), hand‐foot skin reactions (17), fatigue (10), increased AST (12), diarrhea (10)	Advanced HCC patients who have failed prior first‐line therapy	[227]
Ramucirumab	VEGFR2	Phase III RTC (ramucirumab vs. placebo)	NCT02435433	mOS 8.5 vs. 7.3 months	Hypertension (13), hyponatremia (6), increased AST (3)	Advanced HCC patients who have failed prior first‐line therapy and AFP ≥400 ng/mL	[232]
Apatinib	VEGFR2	Phase III RTC (Apatinib vs. placebo)	NCT02329860	mOS 8.7 vs. 6.8 months	Hypertension (28), hand‐foot skin (18), decreased platelet count (13)	Advanced HCC patients who have failed prior first‐line therapy	[238]

Abbreviations: AFP, α‐fetoprotein; ALT, alanine aminotransferase; AST, aspartate aminotransferase; mOS, median overall survival.

## 4 IMMUNOTHERAPY IN HCC

The normal immune system can recognize and attack abnormal cells to maintain the body's homeostasis. The antitumor effects are mainly achieved by cellular and humoral immunity. T cells recognize tumor‐associated antigens (TAAs) and are activated as effector T cells, which kill tumor cells by releasing granzyme, perforin, or cytokines.^239^ B cells can produce specific antibodies against tumor antigens and induce corresponding immune response.^240^ However, HCC cells can evade T cell recognition and killing by reducing the expression of tumor surface antigens and overexpressing immune checkpoint molecules that sending negative regulatory signals. It also induces immune cells to secrete immunosuppressive cytokines and activates specific signaling pathways to inhibit antitumor immune responses.^241^, ^242^

The interaction between immune checkpoints and T cell activation signaling pathways can maintain immune homeostasis and regulate immune responses.^243^, ^244^ Studies have shown that overexpression of immune checkpoint molecules such as PD‐1, CTLA‐4, lymphocyte‐activation gene 3, T‐cell immunoglobulin, and mucin‐domain containing‐3 in HCC contributes to T cell exhaustion and CD8+ T cells apoptosis, thereby promoting immune escape.^245^, ^246^, ^247^ PD‐1 can block the transmission of T cell receptor signals and promote antigen‐specific T cell apoptosis, and continuous PD‐1 signaling can lead to T cell exhaustion.^248^ CTLA‐4, when bound to its ligands B7, delivers inhibitory signals to prevent the activation of effector T cells.^249^ PD‐1/PD‐L1 and CTLA‐4 are currently the most extensively studied inhibitory checkpoint molecules and the main targets for immunotherapy of HCC.^250^

Agents targeting PD‐1/PD‐L1 and CTLA‐4 block the negative feedback pathways of the immune system that mediate immunosuppression, indicating that ICIs have the potential to be an effective treatment strategy. ICIs is currently approved for clinical application, in addition, combination of targeted therapy with ICIs becomes the major treatment, especially antiangiogenesis therapy combined with ICIs.

### 4.1 ICIs monotherapies

Since the approval of the first CTLA‐4 inhibitor ipilimumab for the treatment of metastatic melanoma in 2010,^251^ ICIs have brought new direction for tumor therapy and have shown powerful antitumor effects in HCC. Tremelimumab is the first CTLA‐4 inhibitor for HCC, and has relatively safety and antitumor efficacy in patients with advanced liver cancer and chronic HCV infection (ClinicalTrials.gov, NCT01008358).^17^ Based on phase 1/2 clinical trials CheckMate‐040 (ClinicalTrials.gov, NCT01658878)^252^ and KEYNOTE‐224 (ClinicalTrials.gov, NCT02702414),^253^ PD‐1 inhibitors nivolumab and pembrolizumab have been approved by the US FDA for second‐line treatment of HCC.^254^ However, in subsequent phase 3 clinical trials, CheckMate‐459 (ClinicalTrials.gov, NCT02576509) for nivolumab and KEYNOTE‐240 (ClinicalTrials.gov, NCT02702401) for pembrolizumab failed to meet their expected endpoints, but both had significant OS benefits.^255^, ^256^ The survival benefits of the above monotherapy of ICIs are not very satisfactory for patients with HCC, the combination therapy model based on ICIs is making breakthroughs.

### 4.2 ICIs combinations

According to the data from CheckMate‐040 cohort 4, combination therapy with ipilimumab and nivolumab has been approved by the US FDA for the treatment of patients with HCC who previously had been treated with sorafenib.^257^ Results of the HIMALAYA phase III trial (ClinicalTrials.gov, NCT03298451) showed that tremelimumab plus durvalumab reduced mortality risk and achieved a clinically meaningful OS benefit in patients with HCC.^258^ Consequently, the US FDA approved tremelimumab and durvalumab as first‐line treatment for adults with unresectable HCC.^259^ The strategy of combining inhibitors of different immune checkpoints such as PD‐1 and CTLA‐4 is clinically beneficial against advanced HCC. Other combination strategies are currently being explored.

### 4.3 ICIs combined with targeted drugs

Signal transduction of the immune microenvironment in HCC can also lead to immune evasion. For example, T cell‐derived interferon γ (IFNγ) can promote tumor FGF2 signal transduction, reduce nicotinamide adenine dinucleotide (NAD^+^), activate β‐catenin acetylation, and reprogram tumor dormancy.^260^ The β‐catenin signaling pathway can also downregulate the expression of natural killer group 2D (NKG2D), an NK cell receptor activated by immune cells, thereby reducing the recruitment of inflammatory cells and resulting in poor prognosis for HCC.^261^ VEGF induces abnormal tumor blood vessels, leading to hypoxia and low pH in the TME, which promotes immune suppression.^262^ Overexpression of VEGF inhibits antigen presentation by dendritic cells (DCs), thereby suppressing T cell activation. At the same time, it promotes the recruitment and proliferation of immune inhibitory cells, such as Treg cells and myeloid‐derived suppressor cells (MDSCs), weakening the antitumor immune response.^263^ TGF‐β can inhibit the cytotoxicity of NK cells and the production of related cytokines, as well as suppress antigen presentation by DCs.^264^ TGF‐β collaboratively inhibits the expression of IFN‐γ, thereby inhibiting antitumor activity of CD8^+^ T cells.^265^ FGF19/FGFR‐mediated ETV4 increases the expression of PD‐L1 and chemokine CCL2 in HCC, leading to the accumulation of tumor‐associated macrophages (TAMs) and MDSCs, suppression of CD8^+^ T cell activity, and promotion of HCC metastasis.^266^ Blockade of the STAT3 signaling pathway dramatically enhances the sensitivity of liver cancer cells to NK cell cytotoxicity by upregulating NKG2D ligands.^267^ Since signaling pathways are also involved in immune regulatory mechanisms, the synergies can be created by combining ICIs with targeted drugs.

The researches of Study 117 (lenvatinib combined with nivolumab, ClinicalTrials.gov, NCT03418922) and the KEYNOTE‐524 (lenvatinib combined with pembrolizumab, ClinicalTrials.gov, NCT03006926) both confirmed that ICIs combined with lenvatinib had a better tumor response in first‐line therapy of HCC.^268^, ^269^ More encouragingly, based on IMbrave150, a randomized phase 3 trials (ClinicalTrials.gov, NCT03434379), the US FDA and NMPA approved atezolizumab in combination with BVZ in 2020, for the treatment of unresectable HCC patients who have not previously received systemic therapy.^221^ Following the positive outcome of the IMbrave150, the strategy of combining a PD1/PD‐L1 inhibitor with a VEGF inhibition is considered as a new paradigm for the therapy of advanced HCC. A phase 3 clinical trial ORIENT‐32 (ClinicalTrials.gov, NCT03794440) conducted in China showed that sintilimab combined with IBI305 (a BVZ analogue) demonstrated significant OS and PFS benefits in untreated HBV‐associated HCC patients.^270^ On the basis of the study, the NMPA approved sintilimab in combination with IBI305 for first‐line treatment of unresectable or metastatic HCC in June 2021. Phase 1 (ClinicalTrials.gov, NCT02942329)^271^ and phase 2(ClinicalTrials.gov, NCT03463876) ^272^ trials of camrelizumab in combination with apatinib have demonstrated well antitumor efficacy and reasonable safety profile in patients with advanced HCC. In the phase 3 trial CARES‐310 (ClinicalTrials.gov, NCT03764293),^273^ camrelizumab combined with apatinib significantly improved PFS and OS. The NMPA also approved the combination of camrelizumab and apatinib in 2023 for the first‐line treatment of patients with unresectable or metastatic HCC. Checkpoint inhibitor combined with BVZ has opened a new era in the treatment of HCC, and now, many phase 3 clinical studies of first‐line immunotherapy for liver cancer are being carried out. In the future, combination therapy will continue to be the main direction of HCC.

Clinically, the adverse events of ICIs include autoimmune endocrine diseases, colitis, hypophysitis, hepatitis, pneumonia, and so on.^274^ The skin, gastrointestinal tract, liver, lung, and endocrine organs are most commonly involved.^275^ For CTLA‐4 inhibitors, the incidence of AEs varies with dose, whereas for PD‐1/PD‐L1 inhibitors, the overall incidence of AEs is generally dose independent.^276^ Patients receiving combination immunotherapy have an increased risk of immune‐related adverse events and a higher frequency of multiple organ damage than patients receiving monotherapy (Table 2).^277^

**TABLE 2:** Clinical trials of ICIs for the treatment of advance hepatocellular carcinoma from 2010 to 2023.

Drugs	Drug Type	Stage	NCT number	ORR (%)	DCR (%)	mPFS (months)	mOS (months)	TRAEs (%)	References
Monotherapy									
Tremelimumab	Anti‐CTLA‐4	Phase 2	NCT01008358	NA	76.4	6.5	8.2	45.0	[17]
Nivolumab	Anti‐PD‐1	Phase 1/2	NCT01658878	15/20	58/64	3.4/4.0	15.0/NR	25.0	[251]
		Phase 3	NCT02576509	NA	NA	NA	16.4	49.6	[256]
Pembrolizumab	Anti‐PD‐1	Phase 2	NCT02702414	17.0	62.0	4.9	12.9	25.0	[253]
		Phase 3	NCT02702401	18.3	62.2	3.0	13.9	52.7	[255]
ICIs combinations									
Nivolumab + ipilimumab	Anti‐PD‐1 + Anti‐CTLA‐4	Phase ½	NCT01658878	31.0	49.0	NA	22.8	2.1	[257]
Durvalumab + tremelimumab	Anti‐PD‐1 + Anti‐CTLA‐4	Phase 3	NCT03298451	20.1	60.1	3.8	16.4	75.8	[258]
ICIs combined with targeted drugs									
Atezolizumab + bevacizumab	Anti‐PD‐1 + Anti‐VEGF	Phase 3	NCT03434379	27.3	NA	6.8	NR	61.1	[221]
Pembrolizumab + lenvatinib	Anti‐PD‐1 + TKIs	Phase 1	NCT03006926	46.0	NA	9.3	22.0	67.0	[268]
Nivolumab + lenvatinib	Anti‐PD‐1 + TKIs	Phase 1	NCT03418922	76.7	96.7	NA	NA	NA	[269]
Sintilimab + IBI305	Anti‐PD‐1 + Anti‐VEGF	Phase 2/3	NCT03794440	NA	NA	4.6	NR	14.0	[270]
Camrelizumab + apatinib	Anti‐PD‐1 + TKIs	Phase 3	NCT03764293	33.1	78.3	5.6	22.1	24.0	[273]

Abbreviations: DCR, disease control rate; ICIs, immune checkpoint inhibitors; mPFS, median progression free survival; NA, not available; NR, not reached; ORR, objective response rate; TRAEs, treatment‐related adverse events.

ICIs have entered the clinic and become a valuable treatment choice for solid tumors, further clinical studies are needed to verify the feasibility of ICIs as the main therapeutic for HCC. However, it is clear that immunotherapy is not beneficial in all patients. As more data about these treatments appear, personalized therapy for HCC may be the major trend.

Immunotherapy provides more options for the treatment of HCC. In addition to ICIs, chimeric antigen receptor (CAR)‐T cell therapy is a new type of immunotherapy that specifically kill tumor cells. CAR is a kind of recombinant receptor that binds antigen without major histocompatibility complex (MHC) restriction and guides T cells to recognize and eliminate cells expressing specific target antigen.^278^, ^279^ T cells can recognize TAAs of HCC, such as AFP,^280^ Glypican‐3‐specific (GPC3),^281^ and Mucin 1 (MUC1).^282^ Studies have shown that these antigens can participate in tumor‐specific T cell responses.^283^ CAR‐T targeting TAAs and neoantigen are currently being developed for clinical applications.^284^, ^285^

CAR‐T therapy has been shown to have a good antitumor effect on hematological tumors.^286^, ^287^, ^288^ To develop CAR‐T therapy for solid tumors, antigens that are specifically expressed on the surface of cancer cells need to be explored, and GPC3 may serve as a promising antigen for generating CAR‐T cells targeting HCC.^289^ GPC3, a member of the heparan sulfate (HS) proteoglycan family, is attached to the surface of the cell membrane.^290^ GPC3 is specifically upregulated in HCC, making it a potential therapeutic target for HCC.^291^, ^292^ Studies have shown that GPC3‐targeted CAR‐T cells can induce sustained tumor regression in mice with HCC.^293^ The level of GPC3 expression can also be used as an indicator for the antitumor effect of GPC3‐targeted CAR‐T cells.^294^ Two sequential phase 1 clinical trials (ClinicalTrials.gov, NCT02395250, ClinicalTrials.gov, NCT03146234) have observed antitumor activity of CAR‐GPC3 T cells in HCC patients and demonstrate that the treatment is feasible with preliminary safety.^295^ Li et al.^296^ established dual‐target CAR‐T cells that target GPC‐3 and PD‐1 and showed stronger tumor inhibitory effect on HCC compared with single‐target CAR‐T cells. In addition, disrupting PD‐1 expression through Cas9/CRISPR can improve the anti‐HCC effect of CAR‐T cells in vitro and in vivo, suggesting the potential advantages of CAR‐T cells combined with ICIs in controlling solid tumors.^297^

## 5 CONCLUSION

HCC is a complex disease regulated by multiple signaling pathways, and its occurrence and development are affected by many variables. HCC progression is rapid, and the overall prognosis is poor. Activation or inhibition of signaling pathways and immune escape plays an important role in the development of HCC. Targeted drugs can inhibit tumorigenic receptors and their downstream signaling molecules, and ICIs can block the negative feedback pathway of the immune system that mediates immune suppression. Therefore, the combination of targeted drugs and ICIs appear to be an effective treatment for HCC.

However, due to the high heterogeneity of HCC, different patients or even different tumor lesions in the same patient have different molecular characteristics. So it is difficult for single‐target therapy to be effective for all patients. In this context, genetic detection technology is used clinically to provide precise treatment for patients and drugs combination or multitargeted drugs are recommended. At the same time, HCC patients after targeted drugs treatment can emerge secondary mutations, or change the affinity between targeted drugs and targets, or reactivate downstream signaling pathway conduction to produce off‐target effects and drug tolerance. Therefore, we need to further understand the drug resistance mechanisms related to HCC and find targets that can inhibit multiple signaling pathways simultaneously. In addition, there is still a lack of effective biomarkers to predict the sensitivity of targeted therapy for HCC. Searching for effective biomarkers can better guide the selection of clinical drugs and improve the therapeutic effect.

In recent years, significant progress has been made in immunotherapy for HCC, but the treatment tolerance reduces the effect of ICIs. Relevant studies have shown that ORR of ICIs monotherapy is low, and OS is not significantly improved.^298^, ^299^ Meanwhile, immunotherapy may also lead to overactivation of the immune system to attack its own tissues and organs, resulting in serious AEs, such as immune pneumonia, immune myocarditis, and immune thyroiditis. How to improve the effect of immunotherapy in HCC and reduce its AEs is urgently needed to be resolved.

Currently, the combination of targeted drugs and immunotherapy has been applied in clinical practice and is also a major direction for future research. As mentioned above (the signal transduction of the immune microenvironment in HCC), inhibition of target signaling molecules can activate antitumor immunity in addition to tumor suppression. The commonly used antiangiogenic targeted therapy can normalize tumor blood vessels, enhance the infiltration of immune effector cells into tumors, and synergistically activate the antitumor immune response with immunotherapy. In addition, multikinase inhibitors that target FGFR, PDGFR, and RET are also involved in immune activation.^300^ Therefore, the combination of drugs targeting these pathways with ICIs may achieve synergistic therapeutic effects. Researchers are still committed to exploring new HCC biomarkers, developing more effective molecular inhibitors and ICIs, and investigating novel treatment strategies such as PROTAC and CAR‐T therapy. PROTAC achieves targeted protein degradation in tumors, inhibiting protein activity and reducing the occurrence of drug resistance.^301^ These are currently innovative and promising precision treatment strategies.

This review summarizes the molecular regulatory networks of different signaling pathways in HCC, the relationship between signaling cascades and immune microenvironment, as well as the targeted drugs and ICIs for clinical application in HCC. It provides a clearer understanding of the molecular and immune mechanisms of HCC and will be useful for exploring and developing new targeted drugs and immunotherapy, for guiding selection of more effective synergistic treatments.

## AUTHOR CONTRIBUTIONS

X. L. and X. H. performed the collection of data, and wrote the original manuscript. X. Z. and Y. Z. are responsible for completing the figures and tables. X. Z. are responsible for reviewing the literature. S. H. and Y. S. are responsible for the revision and review of the manuscript. All authors read and approved the final manuscript.

## CONFLICT OF INTEREST STATEMENT

The authors declare no conflict of interest.

## ETHICS STATEMENT

Not applicable.

## Data Availability

Not applicable.
